# Personalized Ultrafractionated Stereotactic Adaptive Radiotherapy in Lung Cancer and Lung Metastases: A Systematic Review

**DOI:** 10.3390/cancers18183021

**Published:** 2026-09-17

**Authors:** Ali Zidan, Gregory W. Chai, David Chen, Adham El-Sherbini, Yee Ung, Michael C. Tjong, Patrick Cheung, Alexander V. Louie, Ian Poon, May N. Tsao

**Affiliations:** 1 Medical Doctor Program, Temerty Faculty of Medicine, University of Toronto, Toronto, ON M5S 1A1, Canada; ali.zidan@mail.utoronto.ca (A.Z.); gregory.chai@mail.utoronto.ca (G.W.C.); davidc42@student.ubc.ca (D.C.); adham.elsherbini@mail.utoronto.ca (A.E.-S.); 2 Division of Radiation Oncology, Department of Surgery, University of British Columbia, Vancouver, BC V5Z 4E6, Canada; 3 Department of Radiation Oncology, Odette Cancer Centre, Sunnybrook Health Sciences Centre, University of Toronto, Toronto, ON M4N 3M5, Canada; yee.ung@sunnybrook.ca (Y.U.); michael.tjong@sunnybrook.ca (M.C.T.); patrick.cheung@sunnybrook.ca (P.C.); alexander.louie@sunnybrook.ca (A.V.L.); ian.poon@sunnybrook.ca (I.P.)

**Keywords:** personalized ultrafractionated stereotactic adaptive radiotherapy, PULSAR, lung cancer, lung metastases, stereotactic body radiotherapy, adaptive radiotherapy

## Abstract

Background/Objectives: Personalized ultrafractionated stereotactic adaptive radiotherapy (PULSAR) delivers high-dose stereotactic radiation as temporally separated pulses, permitting interval response assessment, adaptive replanning, and integration with systemic therapy. Its role in lung cancer and lung metastases remains uncertain. This systematic review synthesizes evidence for PULSAR and PULSAR-like approaches in these settings. Methods: Embase, MEDLINE, and Cochrane Central were searched from inception to 27 May 2026. Eligible records included clinical studies, trial registries, dosimetric simulations, and preclinical studies. Evidence was stratified into five tiers, with lung-target clinical studies considered direct evidence. Design-appropriate tools were used to assess risk of bias, and certainty was evaluated using a GRADE-informed approach. Results: Fifteen records were included: five lung-target clinical reports, one brain-metastasis clinical report, one patient-derived dosimetric simulation, five trial registry records, and three preclinical or computational modelling reports. Clinical evidence comprised small cohorts, case reports, and abstracts. True PULSAR regimens used three to five high-dose pulses separated by three- to four-week intervals with CBCT- or MRI-guided adaptive replanning. Studies suggested technical deliverability, interval tumour regression, and organ-at-risk dose reductions. One lung cohort reported 100% 1-year local control, but comparative safety and efficacy were not established. Pulmonary-function and quality-of-life data were sparse. Preclinical studies supported schedule-dependent interactions between pulsed radiation and PD-L1 blockade. Risk of bias was high to critical, and certainty was very low overall. Conclusions: PULSAR is technically deliverable in high-risk patients, but evidence remains preliminary. Prospective comparative trials are needed to define optimal schedules, adaptation triggers, safety, quality of life, and patient selection.

## Simple Summary

Lung cancer is the most common cancer worldwide, and many patients are too unwell for surgery. Focused, high-dose radiation is often used instead, but this can damage healthy lungs and nearby organs, especially in people with existing breathing problems. A newer approach spaces these high-dose radiation treatments several weeks apart instead of giving them over a few days. The pause lets doctors rescan the tumour, see how much it has shrunk, and redesign the treatment before the next dose. It may also give healthy tissue time to recover and allow the immune system to respond. We systematically reviewed all published studies of this approach for lung tumours to see whether it is practical, safe and effective. The evidence is limited and of very low certainty; early studies suggest that PULSAR can be delivered in these patients, but its effects on tumour control and side effects remain uncertain and require confirmation in larger prospective trials.

## 1. Introduction

Lung cancer is the most frequently diagnosed cancer worldwide and is anticipated to cause more deaths in 2026 than second-ranking colorectal cancer and third-ranking pancreatic cancer combined [1]. The high mortality is largely driven by late-stage presentation: 50% of lung cancers are diagnosed at stage IV, where 5-year survival is only 6%. Only 19% of cases are caught at stage I, where survival is 67%, a more than 10-fold difference [1].

**Figure 1:**
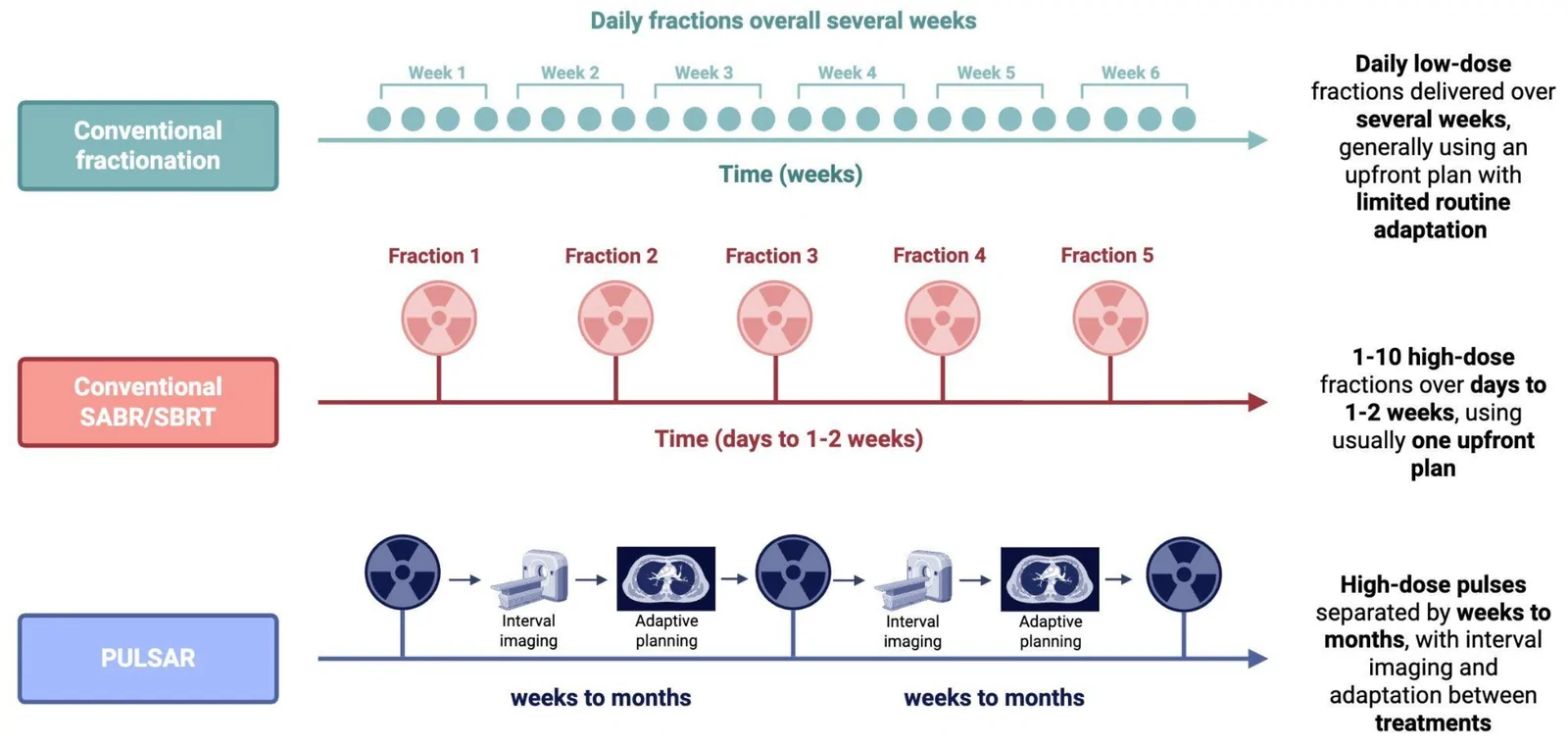
Temporal differences between conventional fractionation, SABR/SBRT, and adaptive PULSAR radiotherapy.

Personalized ultrafractionated stereotactic adaptive radiotherapy (PULSAR) is an emerging radiotherapy paradigm in which stereotactic ablative radiation is delivered as large, temporally separated “pulses” rather than as consecutive or near-consecutive daily fractions [2,3]. Unlike conventional stereotactic body radiotherapy (SBRT) or stereotactic ablative radiotherapy (SABR), in which individual treatments are commonly delivered daily or every other day over a short overall course with limited opportunity for interval adaptation, PULSAR intentionally extends the treatment course by separating ablative pulses over weeks or months [2,3]. The ultrafractionated course in PULSAR refers to a small number of unusually large (ablative fractions) delivered at extended intervals. This temporal separation creates an opportunity for tumour, tumour microenvironment, adjacent normal tissue and systemic host changes to occur between treatments. These interval changes can then be assessed and, when clinically relevant, used to guide adaptive replanning based on tumour response or progression, anatomy, organ-at-risk (OAR) relationships, tolerance, and coordination with systemic therapy [2,4,5]. Differences between PULSAR, conventional SBRT, and conventional fractionation are outlined in Figure 1.

Lung cancer is an ideal setting to study PULSAR due to the following clinical reasons: SBRT is already a standard of care for early-stage lung cancer; lung cancers are visible on imaging; lung cancer targets can change during treatment, making them well suited for adaptive radiotherapy; numerous nearby organs at risk may be spared with adapting the radiation treatment plan due to anatomic changes (particularly with shrinkage of the radiation target); and selected lung cancer metastases are commonly treated with SBRT. Furthermore, there may be possible synergy between immunotherapy and PULSAR, given the established role of immune checkpoint inhibitors in the management of selected patients with non-small cell lung cancer [6,7].

The potential advantages of PULSAR are multifactorial. First, separating ablative radiation pulses may allow normal tissues additional time for repair between treatments, which may be particularly important when treating tumours near critical OARs [8]. Second, extending treatment over time allows for meaningful tumour shrinkage and anatomical changes to be incorporated through adaptive replanning at subsequent fractions, potentially improving dosimetry. Third, the interval between radiation pulses may facilitate immune priming, potentially enhancing the synergy between radiotherapy and immunotherapy and improving anti-tumour responses. Finally, this approach enables in vivo assessment of tumour radiosensitivity, allowing the total radiation dose to be tailored according to response, with fewer planned pulses for patients who achieve an early complete response [8,9,10,11]. These biological and technical features position PULSAR as a potentially more personalized form of stereotactic radiotherapy rather than simply a prolonged version of SBRT.

Although PULSAR remains early in clinical development, its use and investigation have increased in the published medical literature. Initial preclinical work evaluated PULSAR-style radiation in combination with ICB in murine tumour models, demonstrating that radiation sequencing and pulse spacing could influence tumour control [3,12]. Subsequent related studies have expanded this concept through pulsed radiation models, mathematical simulations, and transformer-based approaches designed to characterize the temporal interactions among radiation timing, immunotherapy, tumour response, and immune dynamics [13,14].

Clinically, the literature has progressed from conceptual and mechanistic descriptions to early case reports, institutional experiences, and proposed clinical trials evaluating PULSAR or related split-course SBRT approaches, suggesting the potential emergence of a new era of personalized radiotherapy.

**Figure 2:**
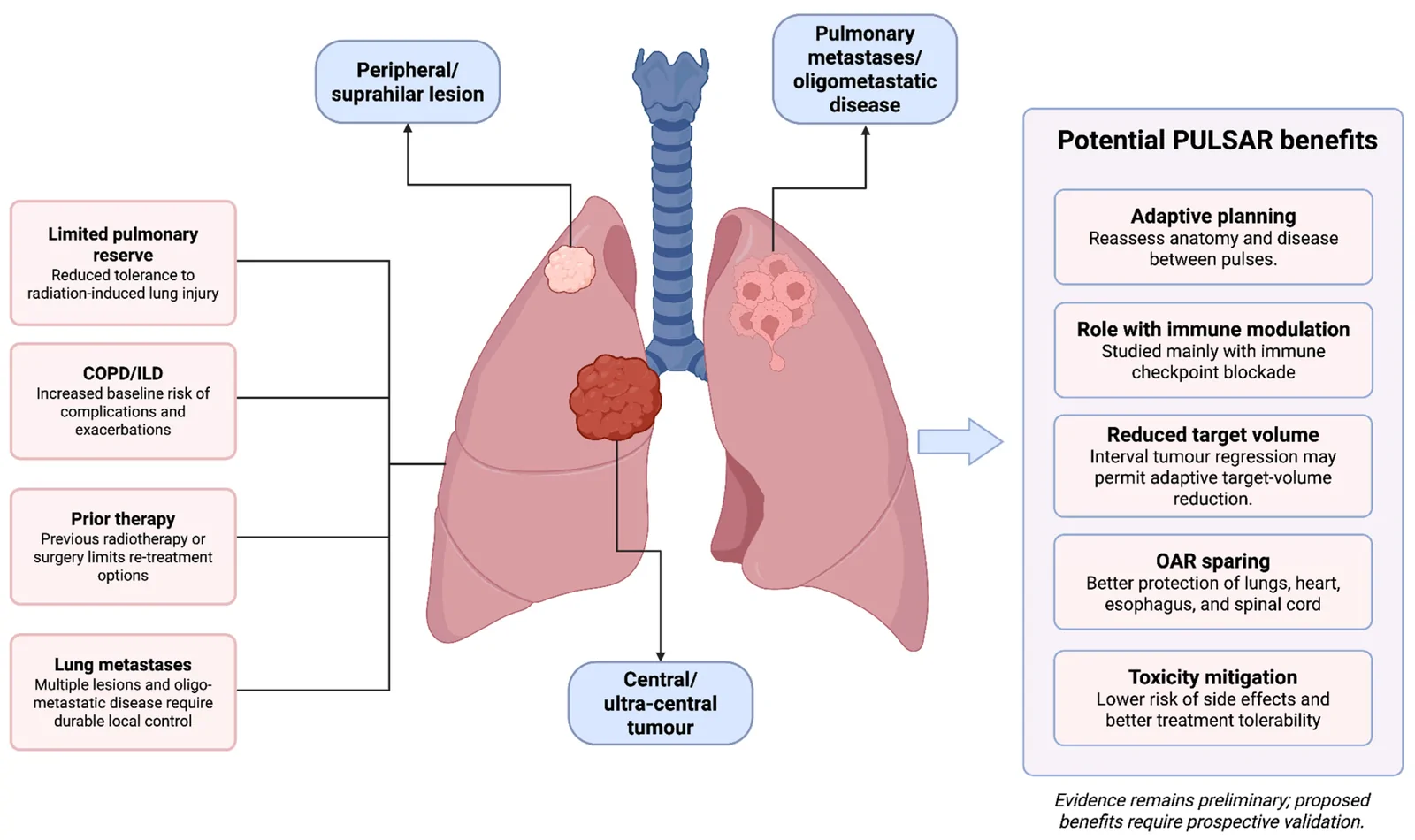
Potential clinical indications and proposed benefits of PULSAR for lung tumours.

Patients with early-stage non-small cell lung cancer (NSCLC), central lung tumours, limited lung metastases, interstitial lung disease (ILD), chronic obstructive pulmonary disease, poor pulmonary reserve, or other factors rendering them medically inoperable may face increased risks from conventional SBRT despite its established role in medically inoperable diseases. In this context, PULSAR has been proposed as a strategy to preserve the ablative intent of SBRT while mitigating toxicity risk through treatment spacing, online adaptation, and response-guided modification of target volumes and OARs [5,10,15]. These potential benefits are outlined in Figure 2.

Important uncertainties therefore remain given the nature of existing studies. The optimal pulse interval, dose per pulse, total dose, criteria for adaptive replanning, management of complete radiographic response during treatment, risk of interval tumour progression, and ideal sequencing with immunotherapy or other systemic agents are not yet established. In addition, the extent to which preclinical immune synergy translates into clinical benefit for patients with lung cancer or lung metastases remains unclear. Given the rapid emergence of PULSAR-related preclinical, clinical, and trial-based literature, this review aims to consolidate the current evidence specific to lung PULSAR.

The objective of this review is to synthesize outcomes such as feasibility, safety, toxicity, pulmonary function, tumour response, local control, survival, adaptive planning, and systemic therapy integration for patients with primary lung cancer or lung metastases receiving PULSAR or PULSAR-like temporally separated stereotactic regimens, while identifying the key translational barriers and research priorities required to guide future clinical implementation.

## 2. Methods

This systematic review was reported in accordance with the Preferred Reporting Items for Systematic Reviews and Meta-Analyses (PRISMA) 2020 guidelines, but as no meta-analysis was conducted, the qualitative synthesis was performed in accordance with the Synthesis Without Meta-analysis (SWiM) guideline (Table S1) [16,17]. A comprehensive search of MEDLINE, Embase, and the Cochrane Central Register of Controlled Trials (Central) was conducted from database inception to 27 May 2026. Keywords searched included lung cancer, radiotherapy, and extended interval dosing. The search strategy is provided in Table S2. Furthermore, the reference lists of the included studies were searched to identify any possible studies that were not included in the search. This study’s protocol was registered on PROSPERO (CRD420261411793, Date of registration 19 June 2026).

All original study designs, including randomized and non-randomized clinical studies with or without comparators, preclinical in vivo studies, and dosimetric or computational studies using patient-derived data or data generated through in vivo experiments, were eligible. Studies were excluded if they were purely theoretical in silico models that were not based on empirical patient-derived or in vivo experimental data, did not involve lung cancer or a relevant metastatic setting, lacked a PULSAR or related high-dose pulsed radiotherapy intervention, or were publications without original data, such as reviews, editorials, and commentaries. The absence of reported clinical outcomes alone was not an exclusion criterion. Title and abstract screening, followed by full-text screening, was conducted independently and in duplicate (AZ, GWC) using Covidence (https://support.covidence.org/, Veritas Health Innovation, Melbourne, Australia). Disagreements were resolved through discussion, with a third reviewer (MNT) acting as adjudicator where necessary. In the case of studies with the same population, studies were only included if they contributed unique data or analyses and the population was counted only once in the final synthesis.

Data extraction was conducted by the same reviewers using a standardized form in Microsoft Excel (Microsoft Corporation, Redmond, WA, USA). Domains extracted included basic study details (such as authors, publication year, and study design), patient baseline characteristics, clinical indications, treatment characteristics, feasibility, safety/toxicity, pulmonary function, dosimetry, adaptive replanning, tumour response, survival, quality of life, and integration with immunotherapy or other systemic therapies.

As the eligible literature spans various populations and study designs, included studies were stratified into five evidence tiers and synthesized separately: (1) clinical studies of patients treated with radiation to the lung such as primary lung cancer and lung metastases, (2) clinical studies reporting outcomes for non-lung studies, (3) dosimetric or in silico planning studies using patient data, (4) clinical trial registries, and (5) preclinical studies. Only (1), clinical studies of patients treated with radiation to the lung, was used as direct evidence for PULSAR.

Risk of bias was assessed at the study level independently and in duplicate by the same reviewers. Non-randomized studies of interventions reporting patient outcomes were appraised using the Risk Of Bias In Non-randomized Studies of Interventions (ROBINS-I) tool across its seven domains [18]. Retrospective cohort studies that report single-arm results with no effect estimates were appraised as an uncontrolled case series using a method described by Murad et al., namely selection, ascertainment, causality and reporting [19]. Case reports were appraised with the Joanna Briggs Institute (JBI) critical appraisal checklist for case reports [20]. Preclinical in vivo studies were appraised with SYRCLE’s risk of bias tool for animal studies [21]. No validated appraisal instrument exists for in silico dosimetric planning studies or for trial registries and these studies were not appraised. Certainty of evidence was appraised for each prespecified outcome using a GRADE-informed narrative approach consisting of the observational design of the available evidence and considering risk of bias, inconsistency, indirectness, imprecision and likelihood of publication bias [22]. Given the heterogeneity of study designs and outcome measures, the review focused on a narrative synthesis to map out existing evidence.

## 3. Results

### 3.1. Study Characteristics

**Figure 3:**
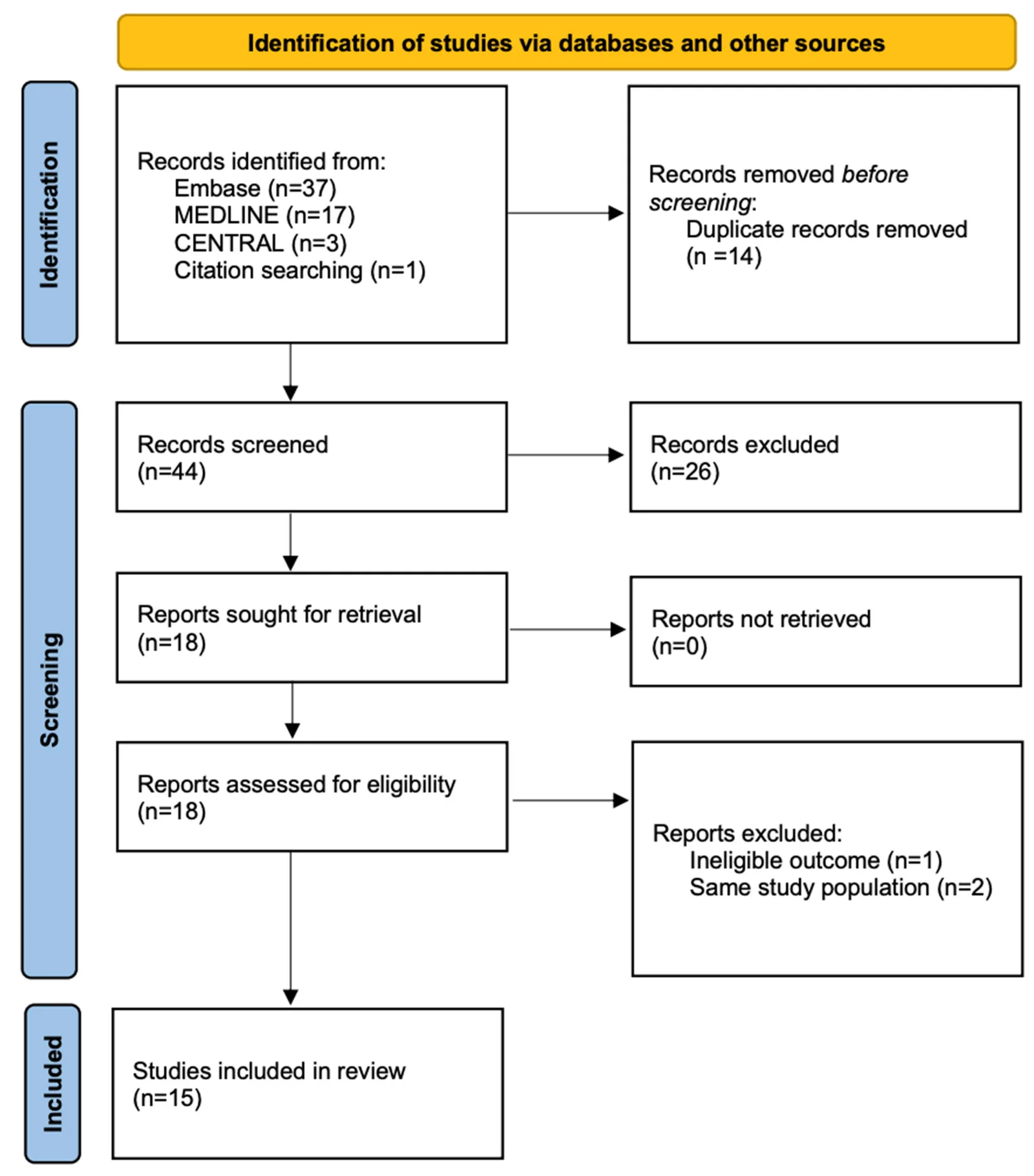
PRISMA flow diagram of study selection.

The database search identified 58 studies in total, with 44 studies screened after removing duplicates (Figure 3). A total of 18 studies were retrieved for full-text screening with 15 studies ultimately being included. Of the 15 included studies, they were composed of seven clinical studies reporting on human participants, five clinical trial registry records, and three preclinical studies conducted in murine models. All records originated from groups based in the United States, except for one case report from Croatia.

It should also be noted that two of the preclinical records drew from the same underlying experimental population as a third preclinical study [3,8,23]. These three records were retained as separate included studies as each study contributed unique content: Moore et al. [3] reported in vivo radiobiological and immunological experiments in a murine cancer model (Lewis lung carcinoma, LLC). The two Peng et al. [8,18] publications reported distinct artificial intelligence modelling approaches. Specifically, a Long Short-Term Memory Recurrent Neural Network (LSTM-RNN) and a transformer model were reported from the same murine cancer model, LLC, used by Moore et al. [3,8,23]. To avoid double counting the current evidence base, their shared population is made explicit when describing study characteristics, as detailed below.

The 15 included articles were published or last updated between 2021 and 2026, with the majority appearing in 2024 (*n* = 6) and 2025 (*n* = 5). The rest of the studies were from 2026 (*n* = 2), 2021(*n* = 1) and 2023 (*n* = 1). By design, these corresponded to two retrospective clinical cohorts, three case reports, one retrospective comparative review, one in silico dosimetric simulation, five interventional trial records, and three preclinical murine experiments (two of which incorporated artificial intelligence modelling). Records were classified as clinical studies if they were published reports of outcomes from treated patients, and as clinical trial records if they were registry entries describing a registered protocol, regardless of whether partial results had been posted. This resulted in seven clinical studies, five clinical trial registries, and three preclinical models.

Geographically, 14 of the 15 records occurred in the United States, with a single case report from Croatia. Amongst the clinical records, the treated or planned populations were predominantly older adults with early-stage or oligometastatic NSCLC, lung metastases, or brain metastases, frequently selected for substantial pulmonary comorbidity or medical inoperability. The three preclinical records used female C57BL/6J mice bearing colon carcinoma (MC38) or Lewis lung carcinoma (LLC) tumours. General characteristics of all records included are summarized in Table 1. PULSAR was also classified into true PULSAR and PULSAR-like. True PULSAR referred to clinically delivered regimens or trial protocols using high-dose stereotactic pulses separated by multi-week intervals, with interval imaging and adaptive or response-guided replanning before later pulses. PULSAR-like refers to temporally separated high-dose stereotactic delivery that lacks at least one of the defining features of PULSAR. This category includes staged or dose staged stereotactic radiosurgery, split course SBRT, simulated pulsed radiation plans and preclinical pulsed radiation models.

### 3.2. Tier 1: Clinical Studies with Lung Irradiation (Direct Evidence)

There were seven clinical reports on patients treated with PULSAR [5,10,15,24,25,26,27] (Table 2). However, only five clinical studies irradiated a lung target. These ranged from single-patient case reports to retrospective cohorts of 24 patients/25 tumours [5,10,15,27]. One abstract reviewed 80 stage-matched SBRT patients of whom only five received a PULSAR-style regimen [24]. Another abstract [25] reported on dosimetric simulation of five anonymized patient datasets rather than a treated cohort [24,26].

Patients in these studies who were treated with PULSAR to the chest were predominantly older adults (median ages from the early 60s to the early 80s) with early-stage or oligometastatic NSCLC and lung metastases. Many of these study participants had substantial pulmonary comorbidities such as ILD, chronic obstructive pulmonary disease (COPD), or supplemental-oxygen dependence, making them high-risk or medically inoperable for conventional SBRT [5,10,15,24].

#### 3.2.1. Treatment and Adaptation Characteristics

Four studies had a PULSAR regimen with lung irradiation which shared a common delivery framework: three to five high-dose pulses separated by three- to four-week intervals, producing treatment courses of roughly two to three months [5,10,15,27]. Reported dose schedules were 40–60 Gy in three to five pulses for lung targets and 36 Gy in three monthly pulses for a stage IIB primary tumour and for oligometastatic disease [5,10,15,27].

All four studies discussed online adaptive replanning between pulses, performed in 80% of patients in the largest cohort and in every delivered fraction of the case reports, using CBCT or MRI-guided platforms. Online adaptive replanning was consistently associated with OAR dose reductions due to interval tumour regression but was not uniform across organs [5,10,15,27]. Notably, in Leipold et al., the internal target volume fell by 18% and then 28%, and the tracheobronchial tree dose volume fell by 28% and 33%, but the lung volume receiving one third of the constraint dose rose by 5% and then 12%, and one rib volume rose by 78% and then 156% [15].

Adaptation triggers, target coverage objectives, margins and organ-at-risk constraints were reported unevenly across the clinical studies. Westover et al. defined the internal target volume as all positions of the gross tumour volume across the respiratory phases and generated the planning target volume by a uniform 5 mm expansion, requiring that 95% of the planning target volume receives at least 100% of the prescription dose [10]. Online adaptation was also performed at the physician’s discretion based on visible anatomical change on set up CBCT or MRI, and when it was performed, contours were re-delineated and plans were reoptimized while the OAR dose constraints were kept the same [10]. Leipold et al. used an initial reference plan as the optimization and contouring template for automatically generated online adapted plans on the Ethos system, derived the internal target volume from a four-dimensional CT in free breathing, and applied an additional isotropic 5 mm margin to generate the planning target volume [15]. Kuehnle et al. published the maximum-dose OAR constraints of 4690 cGy applied to the lungs, 4501 cGy to the main bronchus, 1894 cGy to the trachea, and 491 cGy to the heart, but this was only for planning [5]. Eustace et al. published the only report where the adaptation was due to a prespecified constraint violation where the cumulative heart constraint of V2400 cGy of no more than 15 cm^3^ was exceeded by the non-adapted scheduled plan at the first fraction (22.12 cm^3^ compared with 4.29 cm^3^ with adaptation), and the single-fraction point dose limit of 1000 cGy was exceeded at each of the three non-adapted fractions [27]. Dohopolski et al. described the adaptation process rather than the planning parameters. Dohopolski et al. repeated MRI before each pulse and then the physician assessed the need for replanning [25].

Two comparative records compared pulsed versus consecutive-fraction or conventional SBRT delivery [24,26]. Concurrent systemic therapy accompanied PULSAR in two publications consisting of pembrolizumab (anti-PD-1) in one and central nervous system (CNS)-active systemic agents (a mix of targeted therapies and immunotherapy, given concurrently and adjuvantly) in the other [25,27]. Two reports delivered PULSAR as a monotherapy because poor pulmonary function precluded systemic treatment [5,15].

#### 3.2.2. Reported Outcomes

Feasibility was assessed across all true-PULSAR reports, with full or planned courses delivered using online adaptation [5,10,15,27]. A recurring observation in three studies was substantial interval tumour regression during the inter-pulse pauses. Leipold et al. reported internal target volume (ITV) reductions of 18% and then 28% across successive pulses [15]. Kuehnle et al. observed a reduction in ITV from 24.2 cm^3^ to approximately 4–5 cm^3^ accompanied by a complete radiographic response in one patient that prompted early termination of subsequent radiation pulses [5]. Westover et al. [10] reported a volumetric response in roughly half of treated tumours [10].

Local control was high in the study by Westover et al., which reported 100% local control at 1 year across the full cohort of 24 patients with 25 lung tumours [10]. Westover et al. also reported favourable survival data with a median progression-free survival (PFS) of 21.1 months with 1-year PFS and OS of 80% and 74% for the 24 patients in the study [10].

High-grade toxicity events were uncommon across studies. Westover et al. recorded six grade 3 events (predominantly dyspnea) and no grade 4–5 events within six months [10]. Eustace et al. described isolated grade 2 pneumonitis at one month following concurrent pembrolizumab [27].

Two studies reported on dosimetric statistics using pulsed and adaptive delivery [24,26]. Rahimian et al. [26] found that simulated pulsing lowered heart and lung doses, while Aliru et al. [24] observed a non-significant diffusing capacity of the lungs for carbon monoxide (DLCO) decline with PULSAR (10.5%, *p* = 0.388) versus a significant decline with conventional SBRT (26.1%, *p* < 0.0001). Beyond the DLCO comparison reported by Aliru et al., pulmonary function was not systematically assessed after treatment in most reports, and no study reported validated quality-of-life outcomes [24,26]. However, only Aliru et al. produced a clinical study with direct evidence while Rahimian et al. produced an in silico study.

### 3.3. Tier 2: Clinical Studies with Non-Lung Targets (Indirect Evidence)

Dohopolski et al. published the one study that reported on the use of PULSAR for brain metastases, most commonly from NSCLC and breast cancer [25]. Dohopolski et al. treated 55 patients with 109 brain metastases using pulsed Gamma Knife radiosurgery at two- to four-week intervals with repeated MRI performed before each pulse; adaptation was up to the physician’s discretion. Concurrent or adjuvant CNS-active systemic agents were used in part of the cohort. Reported outcomes were 1-year and 2-year local failure of 5.0% and 8.9%, a median overall survival that was not reached at a median follow-up of 1.72 years, and a grade 3 or higher vasogenic edema rate was stable at 8.7% at both 1 and 2 years with no significant difference by concurrent CNS-active drug use [25].

### 3.4. Tier 3: Dosimetric and In Silico Planning Studies

One included study reported dosimetric endpoints from simulated treatment. Rahimian et al. [26] modelled adaptive dose-staged delivery for large central NSCLC using patient-derived planning data and found that simulated pulsing lowered heart and lung doses relative to a conventional plan. The entire reported gain is conditional on an assumed uniform PTV reduction after each pulse but was not measured. As the study was only simulated, the study reports no patient outcomes.

### 3.5. Tier 4: Clinical Trial Registries

Five registry records described interventional trials of PULSAR (Table 1), all based in the United States [28,29,30,31,32]. Trial designs included two phase I studies [28,29]. There are three randomised phase II trials in the clinical trials registry [30,31,32].

All five registry trial records described PULSAR treatment designs in which high-dose radiation pulses were delivered at extended intervals, with adaptive or response-guided replanning between pulses [28,29,30,31,32]. Several designs incorporated explicit adaptive features, such as omission of subsequent pulses upon a defined volumetric response [27,28] and per-pulse replanning to reduce dose or fraction number.

Most trials integrated PULSAR with immunotherapy such as durvalumab, pembrolizumab, nivolumab, or standard-of-care PD-L1 inhibitors, reflecting a focus on radiation–immunotherapy combination [28,29,30,31,32]. The planned trial characteristics are summarized in Table 3.

**Table 3:** Clinical trial planning and design characteristics of registered PULSAR trials.

Trial (Author Name + Year of Last Update)	Phase/Design	Status	Planned *N*	Disease/Setting	Primary Objective
Desai 2025 [28]	Phase I multicohort platform, open-label	Recruiting	45	Cohort A: ES-SCLC; Cohort B: brain metastases	Safety/feasibility and preliminary efficacy
Westover 2025 [29]	Early phase I single-arm feasibility	Recruiting	30	Ultra-central lung tumours (primary/metastatic)	Feasibility of MRI-guided adaptive PULSAR
Widhelm 2025 [30]	Phase II RCT (3 + 3 run-in), open-label	Terminated (corporate strategy)	40 (6 enrolled)	Oligometastatic NSCLC and RCC	Progression-free rate at 18 months
Badiyan 2026 [31]	Phase II RCT, open-label	Terminated (funding halted)	52 (1 enrolled)	Metastatic NSCLC	Quality of life (EORTC QLQ-C30) over 2 years
Kumar 2024 [32]	Phase II RCT, open-label	Withdrawn (never initiated)	46 (0 enrolled)	NSCLC with up to 10 brain metastases	Intracranial clinical benefit at 6 months

Among the five registered trials, two were actively recruiting [28,29], one was withdrawn before initiation [32], and two were terminated early [30,31] (Table 2). No trial was recorded as completed.

### 3.6. Tier 5: Preclinical Evidence

Three preclinical records evaluated pulsed radiotherapy in murine tumour models [3,8,23]. It is important to note that these studies assessed PULSAR-like adaptive radiotherapy approaches. These PULSAR-like radiotherapy maintained pulsed dosing schedules and associated computational modelling but did not involve PULSAR treatment in humans. These findings characterize the radiobiological evidence for the PULSAR concept rather than clinical performance.

#### 3.6.1. Preclinical Characteristics

All three preclinical records originated in the United States and used female C57BL/6J mice, 6–8 weeks old, bearing subcutaneous right-leg tumours. Moore et al. [3] employed both murine (mouse) colon adenocarcinoma 38 (MC38) colon carcinoma and LLC models, while Peng et al. [8,23] used the immune-resistant cold LLC model [3,8,23]. Group sizes were generally seven to nine animals per arm. Notably, the animal models from Moore et al. [3] were the source population for both the Peng et al. publications [8,23] re-analyzed and augmented the original experimental data for artificial intelligence modelling [3,8,23].

#### 3.6.2. Preclinical Experimental Models and Design

In all three studies, treatment was initiated once the murine subcutaneous tumours reached a defined volume (100–150 mm^3^ for MC38 and 150–200 mm^3^ for LLC), with radiation delivered using a dedicated kilovoltage X-ray irradiator (250 kVp, 15 mA, dose rate 19.468 Gy/min). PULSAR-style schedules used radiation pulses separated by intervals of 0, 1, 4, or 10 days, with the 10-day spacing identified as the key comparator. Doses ranged from single pulses to two-pulse regimens (e.g., MC38: 2 × 8 Gy; LLC: 2 × 10–15 Gy, or 20 Gy on day 1 plus 20 Gy on day 10). All three studies combined pulsed radiation with programmed death ligand-1 blockade (α-PD-L1), varying the relative timing of immunotherapy and radiation, and Moore et al. [3] additionally performed CD8+ T-cell depletion experiments to test immune dependence. The two Peng et al. publications [8,23] performed computational modelling, an LSTM-RNN recurrent model in one and a transformer-based model in the other, using data augmentation (to approximate 1200–1300 samples) in order to predict tumour-volume trajectories from radiation dose, timing, and α-PD-L1 schedule [8,23].

#### 3.6.3. Preclinical Outcomes Reported

The three preclinical records reported that the potential benefit of combining pulsed radiation with PD-L1 blockade was schedule-dependent rather than a simple additive effect of the two modalities [3,8,23]. In the cold LLC model, α-PD-L1 alone produced no meaningful tumour control. An additive benefit over radiation alone emerged only with 10-day pulse spacing and required the second pulse. Whereas near-daily (1-day) spacing conferred no advantage. Moore et al. [3] further established that tumour control improved when α-PD-L1 was given during or after radiation rather than before, that an upfront single pulse was most effective in the MC38 model, and that Cluster of Differentiation 8+ (CD8+) T-cell depletion reduced or abolished the combination effect, demonstrating CD8+ T-cell dependence. The computational analyses by Peng et al. [8,23] involved in silico experiments, with each model contributing a distinct prediction. The LSTM-RNN model predicted optimal tumour control when anti-PD-L1 administration began 7–8 days after a single 40 Gy radiation pulse. For a regimen of two 10 Gy radiation pulses separated by 10 days, four consecutive daily anti-PD-L1 administrations were predicted to result in poorer tumour control than two consecutive daily administrations. The transformer model identified maximal synergy at 10-day pulse spacing delivered when the second α-PD-L1 dose was given concurrently with the second pulse. These three preclinical studies did not have toxicity as a formal endpoint. The euthanasia criteria (tumour volume >1500 mm^3^, any dimension >2 cm, or significant ulceration) were used to define the survival endpoint. The modelling reports by Peng et al. [8,23] cautioned that several volume-curve interpretations were limited by large error bars and by reliance on augmented rather than directly measured data [3,8,23].

### 3.7. Risk of Bias

Risk-of-bias judgments for all included studies are presented in Table 4. As four different instruments were required, the judgements are reported in each instrument’s own terms and are not interchangeable across designs. Aliru et al. published the only study that reports a comparison of PULSAR against a comparator strategy; they compared five patients receiving a PULSAR-like schedule with 45 receiving conventional SBRT (Aliru 2023 [24]), and that unadjusted comparison is at critical risk of bias. The two retrospective cohorts reporting single-arm results were appraised as uncontrolled series using the tool of Murad et al. [19]. The methodological quality of the reports by Westover et al. [10] and that of Dohopolski et al. [25] was adequate, with reservations regarding selection, as 24 of 177 treated lesions were excluded because of less than four months of follow-up; the evidence was also considered indirect for the review question. In all three, the limiting feature is the design rather than any deficiency of reporting, where the two cohorts have no comparator, and the one comparison that exists is unadjusted.

All three case reports were appraised under the JBI checklist. They were generally well reported with a complete patient history, diagnostic assessment, the intervention, and the takeaway lessons were clearly described in all three. The deductions were an unconfirmed histological diagnosis in one report, in which biopsy was judged unsafe (Kuehnle 2025) [5] and the absence of any post-intervention clinical condition or adverse-event reporting in another, which reported dosimetric endpoints only (Leipold 2024) [15].

Under SYRCLE’s tool, all three preclinical records (Moore 2021; Peng 2024) were at an unclear-to-high risk of bias, the high rating being driven by item seven, the blinding of the outcome assessor [3,8,23]. Baseline characteristics were well controlled, and allocation was stratified by tumour volume, but allocation concealment, random housing, and blinded outcome assessment were not reported, and no preregistered protocol exists. The two modelling records inherit these concerns and add reliance on augmented rather than directly measured data (Peng 2024 [8,23]).

The dosimetric study and the registry records are not gradable, no validated instrument existing for either and neither reporting an observed clinical outcome. Neither can support an estimate of effect. The individual risk-of-bias extraction can be found in Table S3.

### 3.8. Certainty of Evidence

The certainty of evidence was very low for every claim which had supporting evidence (Table 5). No included study compared PULSAR with conventional SBRT with toxicity, local control, survival, or quality of life, so for those comparisons, the evidence is absent rather than uncertain. Publication bias is likely across all outcomes; the only unfavorable patient-level data in this review are the two registry postings, neither of which were later published.

### 3.9. Priority Subgroups for Prospective Clinical Trial Validation

A preliminary, risk-stratified framework was created to identify priority trial populations for PULSAR (Table 6). The subgroups in which a standard five-fraction plan violates a constraint or carries a well-documented excess toxicity risk are prioritized, whereas lower peripheral early-stage disease, for which SBRT already achieves high local control with low toxicity and the added burden of a two- to three-month adaptive course is unlikely to be offset, is lower in priority. No subgroup in this framework is supported by comparative evidence as the ranking reflects where the reviewed evidence indicates a constraint exists.

## 4. Discussion

The evidence supporting the utility of PULSAR has grown from a conceptual extension of SBRT towards a clinical strategy reported in a small number of selected patients but remains in an early, hypothesis-generating stage. PULSAR is defined by temporal separation of ablative pulses, interval response assessment, and adaptive replanning. The contemporary evidence included in this review is small in sample size and heterogeneous in study design and reported outcomes, spanning clinical cohorts, case reports, trial registry records, and preclinical studies [3,5,8,10,15,23,27]. The studies demonstrate that a three-to-five pulse schedule at three- to four-week intervals can be delivered to high-risk patients and that online adaptation is achievable whenever attempted. They indicate that adaptation usually lowers dose to mediastinal organs at risk but at small margins, not uniformly, and with lung dose rising as the target regresses in one report. They do not show that this reduces clinical toxicity, preserves pulmonary function, improves quality of life, or maintains local control relative to SBRT as there are no prospective comparative studies or adjustments for confounding.

PULSAR represents a promising investigational strategy in clinical settings where conventional SBRT may be limited by target coverage or OAR constraints [34,35,36]. We hypothesize that PULSAR may be considered when SBRT schedules yield compromises in prescription dose, target coverage, or OAR constraints. However, this remains a hypothesis as no included study enrolled a patient for which a conventional SBRT plan was deliverable and compared the two approaches.

Lung cancer radiation targets abutting critical OARs such as the proximal bronchial tree, esophagus, heart, great vessels, or brachial plexus can contribute to a higher risk profile compared to peripheral, early-stage disease [33]. PULSAR may also be considered in the non-curative treatment of very bulky thoracic or other tumours, using pulses delivered every few weeks to allow interval tumour regression and potentially achieve more durable local control than conventional palliative radiotherapy. However, as no study compared PULSAR to palliative radiotherapy, this also remains a hypothesis.

The potential benefit for a PULSAR approach is adaptation of target coverage. With shrinkage of the target after each pulse, there may be better OAR sparing. Also, with extended periods between PULSAR fractions, there may be improved repair of radiation-induced injury to normal tissue. For the uninvolved lung, this may result in less lung injuries for patients with COPD or interstitial lung disease. However, this is unproven clinically.

PULSAR schedules were most often delivered in three to five pulses separated by three to four weeks. The largest cohort included in this review was from a study by Westover et al., who evaluated PULSAR treatment in high-risk, early-stage NSCLC patients with substantial pulmonary comorbidities and found that online adaptation of a PULSAR regimen of 40–60 Gy in 3–5 fractions could feasibly yield complete tumour control without grade 4 or 5 adverse events, though six grade 3 events occurred within six months [10].

Online adaptation using CBCT-guided or MRI-guided platforms was reported in most cohorts, with interval imaging of the primary tumour and OAR constraints to revise target coverage [5,10,15,27]. The feasibility of online adaptation during the scheduled interruptions of PULSAR allows dynamic reassessment of clinically actionable changes, such as target displacement, target regression, and changes to surrounding at-risk structures, that can now be readily accounted for in subsequent treatment plans.

However, online adaptive workflows are presently resource intensive. PULSAR requires repeated image acquisition, clinician review, revision of the treatment plan, and quality assurance, which may influence the viability of PULSAR among different radiotherapy centres.

Interval tumour regression is a distinguishing factor observed in studies of PULSAR interventions compared to SBRT with conventional, uninterrupted schedules [37]. In one case report [5], PULSAR was used for a stage I, ultra-central tumour in the setting of multiple pulmonary comorbidities. Marked regression was observed during serial treatments which led to complete radiographic response and discontinuation of the full treatment course [5]. However, regression of this magnitude was substantial, and across the largest cohort, the median tumour retained 70% of its initial volume at maximal response, so these findings should be interpreted with caution, whereby the dosimetric benefit of adaptation should not be generalized from the most responsive of cases.

Preclinical studies provide a biological rationale for combining PULSAR with systemic therapies. These limited data generate the hypothesis that the addition of systemic agents, particularly ICB, may enhance the therapeutic efficacy of PULSAR. One hypothesis is that each ablative treatment leads to tumour cell death which releases antigens used by recruited T-cells to mount an immune response. The synergistic effect of PULSAR and ICB relies on the scheduled interruptions of PULSAR to give time for T-cells to respond to new tumour antigens released with each ablative treatment [3,13,38].

In the Moore et al. preclinical study, pulsed radiation with anti-PD-L1 in the immune-resistant lung carcinoma murine model was more effective with wider pulse spacing and required CD8+ T-cell activity [3]. This finding is consistent with preclinical mechanistic evidence that suggests that radiation can influence immunogenicity, triggering abscopal immune effects by priming CD8+ T-cells to promote systemic tumour rejection [39,40]. Taken together, the preclinical studies support the hypothesis that pulse timing and frequency may play an important role in promoting the synergy between radiation and immunotherapy, warranting future comparative human trials of PULSAR schedules versus the standard of care in selected patients.

There have been early closures of several PULSAR and ICB trials [31,32,41]. Limited patient accrual; complex, adaptive radiotherapy trials requiring multicentre enrollment for accrual; adequate funding to support trial conduct; and staff resources needed for on-treatment adaptation may be significant barriers to the completion of PULSAR trials.

Several limitations to this review include the small sample size of participants treated with PULSAR. There are no large randomized controlled trials that examine the use of PULSAR compared to the standard of care. The published literature consists of case reports, retrospective cohorts, and preclinical studies. Despite these studies generating hypotheses, further high-quality clinical trials are needed to systematically characterize the impact of PULSAR interventions on survival, local control, toxicity, and quality of life in patients. Second, the disease histology and setting of study participants were heterogeneous across included studies, which included primary lung cancer, lung metastases, oligometastatic NSCLC, extensive-stage SCLC, and brain metastases. Third, there was high variability in study-specific protocols regarding PULSAR regimen (dose fractionation, pulse interval), the imaging platform used (CBCT, MR), adaptation trigger, and response criteria. Fourth, publication bias may be present, as studies reporting favourable responses to PULSAR may have been more likely to be published compared to studies where there was little or no treatment effect. Fifth, most of the evidence is indirect where the largest clinical dataset treats an intracranial target; the dosimetric evidence rests on simulated rather than observed regression; and the biological rationale rests on one murine experiment [3,8,23,25,26]. Finally, follow-up in the only cohort reporting local control was a median of 16.2 months, which may have been too short to characterize late toxicity or long-term local control [10].

Future PULSAR studies should prioritize establishing a minimum complete set of reporting items, such as pulse dose, total dose, biological effective dose, inter-pulse interval, overall treatment time, imaging platform, adaptation trigger, dose and contour adaptation protocol, and systemic therapy timing, to ensure that results can be reproduced and critically appraised.

Large prospective trials are needed to examine safety and efficacy outcomes (overall survival, local control). Furthermore, large trials are needed to determine whether certain lung cancer patients are more likely to benefit from PULSAR.

Other important clinical endpoints beyond toxicity, overall survival, and local control rates include DLCO, forced expiratory volume in 1 s (FEV1) decline, supplemental oxygen requirement, dyspnea scores, quality of life, and time to next systemic therapy.

## 5. Conclusions

PULSAR is an emerging radiotherapy strategy that combines high-dose ablative pulses with temporal interruptions, interval response assessment, and adaptive replanning. Current evidence remains preliminary and of very low certainty, derived mainly from small cohorts, case reports, trial registrations, and preclinical studies. What the clinical evidence shows is that three to five pulses at three- to four-week intervals can be delivered to medically inoperable patients. The available clinical evidence suggests that online adaptation is technically feasible and can reduce OAR dose in selected cases; however, the limited sample sizes and absence of comparative studies preclude firm conclusions regarding safety. Moreover, our review does not show any comparative benefit of PULSAR since no included study compared PULSAR with conventional SBRT for toxicity, local control, survival or quality of life, Additional studies are needed to define the optimal scheduling and clinical role of PULSAR. Although early experience is encouraging, prospective clinical trials are needed to determine its impact on local control, overall survival, and quality of life, and to identify the patient subgroups most likely to benefit from this approach.

## Figures and Tables

**Table 1:** General study characteristics of included studies.

Study (First Author + Year)	Design (Publication Type)	Country	Sample Size	PULSAR Fidelity	Category
**Preclinical Studies**					
Moore 2021 [3]	Preclinical murine experiment (original article)	USA	Mice, ~7–9 per group	PULSAR-like (preclinical)	Preclinical
Peng 2024 (LSTM-RNN) [23]	Preclinical murine experiment + AI modelling (original article)	USA	~182–208 mice (26 groups); same experimental population as Moore 2021	PULSAR-like (preclinical)	Preclinical
Peng 2024 (Transformer) [8]	Preclinical murine experiment + AI modelling (original article)	USA	~7–8 mice/group (24 groups); same experimental population as Moore 2021	PULSAR-like (preclinical)	Preclinical
**Clinical Studies**					
Aliru 2023 [24]	Retrospective review (conference abstract)	USA	80 patients (5 PULSAR)	PULSAR-like vs. SBRT/SABR	Clinical
Dohopolski 2025 [25]	Retrospective cohort (original article)	USA	55 patients/109 lesions	PULSAR-like	Clinical
Westover 2026 [10]	Retrospective cohort (original article)	USA	24 patients/25 tumours	True PULSAR	Clinical
Rahimian 2023 [26]	Retrospective in silico dosimetric simulation (abstract)	USA	5 patient datasets	PULSAR-like (simulation)	Clinical (dosimetric)
Leipold 2024 [15]	Single-patient case report	Croatia	1 patient	True PULSAR	Clinical
Kuehnle 2025 [5]	Single-patient case report	USA	1 patient	True PULSAR	Clinical
Eustace 2024 [27]	Single-patient case report	USA	1 patient	True PULSAR	Clinical
**Trial Registries**					
Desai 2025 [28]	Phase I multicohort platform trial registry record	USA	Planned 45 (recruiting)	True PULSAR	Trial registry
Westover 2025 [29]	Early phase I single-arm feasibility trial registry record	USA	Planned 30 (recruiting)	True PULSAR	Trial registry
Widhelm 2025 [30]	Phase II RCT registry record	USA	Planned 40; enrolled 6	PULSAR-like	Trial registry
Badiyan 2026 [31]	Phase II RCT registry record	USA	Planned 52; enrolled 1	PULSAR-like	Trial registry
Kumar 2024 [32]	Phase II RCT registry record	USA	Planned 46; enrolled 0	True PULSAR	Trial registry

**Table 2:** Individual study characteristics, including disease setting, PULSAR delivery, and key reported findings for clinical studies.

Study (First Author + Year)	Cancer Type/ Setting	Lesion Location and Size (*n*)	PULSAR Regimen (Dose, Interval)	Adaptive Replanning	Systemic/ ICB	Key Findings
Westover 2026 [10]; retrospective analysis of consecutively treated patients	Early-stage (T1-T2N0) NSCLC and limited lung metastases; high-risk pulmonary cohort (ILD 29%, COPD 67%)	Primary lung (*n* = 22) and metastatic (*n* = 2); 36% central, 64% peripheral; median diameter 25 mm	40, 50, 54 or 60 Gy in 3–5 pulses; pulses every 3 weeks; median course 83 days	Online adaptive RT in 80% CBCT and MR linac significant OAR dose reductions	None reported	1-year local control 100%; median PFS 21.1 mo; 1-yr PFS 80%, 1-yr OS 74%; 6 grade 3 events, no grade 4–5
Leipold 2024 [15]; case report	NSCLC, stage IIB (cT3N0); medically inoperable, severe COPD	Right upper lobe primary (*n* = 1); enlarged to 71 × 49 mm at simulation	3 pulses of 12 Gy 36 Gy peripheral; D50% 45 Gy every 4 weeks	Online CBCT-based adaptation with each pulse	None (due to poor pulmonary function)	ITV reduced 18% then 28% across pulses; OAR doses reduced with each pulse; no toxicity outcomes reported
Kuehnle 2025 [5]; case report	Presumed early-stage NSCLC (IA3, cT1cN0; no biopsy); scleroderma /ILD on home O_2_	Right suprahilar/right upper lobe mass, *n* = 1 2.6 cm; ITV 24.2 cm^3^	Planned 40 Gy in 5 pulses every 3 weeks; 32 Gy in 4 pulses delivered (stopped early)	Online adaptive (CBCT) each fraction	None	Complete radiographic response prompting early termination; no grade 3+ events; O_2_ sat maintained at 3 months
Eustace 2024 [27]; case report	Oligometastatic NSCLC (T3N3M1b), adenocarcinoma; PD-L1 90%	Left upper lobe primary + nodal disease; *n* = 1 femur metastasis; GTV 103 cm^3^	36 Gy in 3 pulses (12 Gy each) every month (BED 79.2 Gy)	CT-guided online adaptation for thoracic target; femur non-adaptive	Concurrent pembrolizumab	No progression at 6 m; reduced heart and bronchus dose; grade 2 pneumonitis at 1 month
Dohopolski 2025 [25]; retrospective	Brain metastases from solid tumours (NSCLC 37%, breast 28%, melanoma 11%, others)	109 brain lesions; *n* = 55; median volume 4.0 cm^3^; 51 lesions > 2 cm	PULSAR 2 or 5 treatments, 2–4-week intervals; median BED 48 Gy	Repeat MRI between treatments; physician-directed response-adapted replanning	Concurrent and adjuvant CNS-active drugs	1-/2 yr local failure 5.0%/8.9%; 2 yr grade 3+ vasogenic edema 8.7%; median OS not reached
Aliru 2023 [24] (abstract)	Medically inoperable early-stage NSCLC and pulmonary metastases; poor vs. normal lung function	Lung lesions (primary and metastatic); *n* = 5; size N/A	PULSAR subset (*n* = 5): one fraction every 1–3 weeks vs. conventional SBRT	Not reported for PULSAR subset	None reported	DLCO decline −10.5% (PULSAR, *p* = 0.388) vs. −26.1% (SBRT *p* < 0.0001); OS worse with poor lung function on SBRT
Rahimian 2023 [26]; retrospective in silico dosimetry study	Large central NSCLC lung tumours (in silico)	Central lung; *n* = 5; mean ITV 30.0 cc, mean PTV 65.4 cc	5 pulses of 10 Gy vs. 50 Gy in 5 consecutive fractions	Four adaptive PTVs simulated per patient	None	Simulated pulsed plan lowered heart, ipsilateral and contralateral lung dose (11 Gy ipsilateral lung volume, 136 versus 241 cc)

PULSAR = personalized ultrafractionated stereotactic adaptive radiotherapy; ICB = immune checkpoint blockade; CBCT = cone-beam computed tomography; MR = magnetic resonance; *n* = number of patients; BED = biological effective dose; CNS = central nervous system; DLCO = diffusing capacity of the lungs for carbon monoxide; OS = overall survival; SBRT = stereotactic body radiotherapy; Gy = gray; cc = cubic centimetres; ITV = internal target volume; PTV = planning target volume. Note: Clinical trial registry records are summarized separately in Table 3.

**Table 4:** Study risk of bias level.

Study (First Author + Year)	Appraisal Tool	Domains of Concern	Overall Judgment
Westover 2026 [10]	Murad et al. [19]	Causality: no comparator, so outcomes cannot be attributed to PULSAR rather than to selection, natural history, or competing risks. Ascertainment: toxicity graded with CTCAE v5.0 at visits but collected and adjudicated retrospectively and unblinded. Selection and reporting are adequate: consecutively treated patients with defined eligibility, and doses, schedules, and outcomes reported in full.	Adequate with reservations
Dohopolski 2025 [25]	Murad et al. [19]	Selection: of 177 treated lesions, 109 were retained; most exclusions were definitional, but 24 lesions were excluded for less than four months of follow-up. Ascertainment: Retrospective; unblinded imaging-based local failure and toxicity grading; treatment style classified post hoc. Causality: no comparator for PULSAR; the only internal comparison is non-randomized use of CNS-active drugs. Additional indirectness: intracranial target and not lung radiation.	Adequate with reservations
Aliru 2023 [24]	ROBINS-I	No attempt to control confounding: five PULSAR-like patients compared with 45 receiving conventional SBRT without adjustment, over a treatment era spanning 2005 to 2022. Abstract-only reporting. The DLCO estimate has a confidence interval spanning −40.7% to +19.7%.	Critical risk of bias
Leipold 2024 [15]	JBI checklist for case reports	No post-intervention clinical condition and no adverse-event reporting; endpoints are dosimetric only.	Include
Kuehnle 2025 [5]	JBI checklist for case reports	The source reports no treatment-related effects within six months of starting therapy, with a documented visit at three months after the last fraction at which fatigue and asymptomatic radiographic changes were noted.	Include
Eustace 2024 [27]	JBI checklist for case reports	Attribution of the grade 2 pneumonitis between radiation and concurrent pembrolizumab is uncertain and acknowledged by the authors.	Include
Moore 2021, Peng 2024 (LSTM-RNN) and Peng 2024 (Transformer) [3,8,23]	SYRCLE	Investigators are reported to be blinded to allocation during the experiment and drug treatment but only whenever possible. Allocation concealment, random housing, random outcome assessment and blinding of outcome assessment are not reported, and caliper tumour measurement is susceptible to unblinded assessment. There was no preregistered protocol, so selective outcome reporting cannot be excluded.	Report domain-level judgments; no overall summary rating

Note: Rahimian 2023 [26] and the trial registries were not assessed due to having no validated risk-of-bias instrument.

**Table 5:** Certainty of evidence by outcome (GRADE-informed narrative assessment).

Outcome	Evidence	Domain Judgments	Certainty
Feasibility and technical deliverability (a schedule of three to five pulses at three- to four-week intervals can be delivered to medically inoperable, pulmonary compromised patients with online adaptation)	Four uncontrolled lung radiation reports totalling 27 patients	Risk of bias is very serious due to being composed of a single-arm retrospective cohort and case reports; all reports are concordant; indirectness is not serious; imprecision is very serious due to low number of patients; publication bias is suspected due to case reports	Very low
Descriptive analysis of safety and toxicity (grade 3 events occur at the observed frequency in this population)	One lung radiation cohort out of 24 cohorts, two single-patient case reports, and results from participants of registry trials	Risk of bias is very serious due to unblinded data collection; inconsistency is not assessable; indirectness is serious as registry regimens do not isolate PULSAR from concurrent systemic therapy; imprecision is very serious due to low sample size; publication bias suspected due to case reports	Very low
Pulmonary function (PULSAR preserves pulmonary function relative to conventional SBRT)	One conference abstract where five patients received a PULSAR-like schedule	Risk of bias is critical; inconsistency is not assessable due to being a single study; indirectness is serious as the interval varies compared to any true PULSAR; imprecision is serious (DLCO −10.5%, 95–40.7% to +19.7%); publication bias is suspected due to being an abstract	Very low
Tumour response (measurable interval regression occurs between pulses)	One cohort of 24 patients with 25 tumours (Westover et al.) and two patient case reports (Kuehnle and Leopold)	Risk of bias is very serious; inconsistency is serious (complete response to none); indirectness is not serious; imprecision is very serious; publication bias is suspected	Very low
Local control (local control at one year is high in this population)	One uncontrolled cohort with a cumulative incidence of local recurrence of 0% across 24 patients with 25 lung tumours	Risk of bias is very serious; inconsistency is not assessable due to being a single study; imprecision is very serious (single arm); publication bias is suspected	Very low

**Table 6:** Preliminary risk stratified framework for prioritizing subgroups in future PULSAR trials.

Priority	Subgroup	Rationale	Evidence	Suggested Focus
Highest	Central or ultra-central lung tumours (primary or metastatic) in which a standard five-fraction SBRT plan violates an organ-at-risk constraint.	This is the setting in which the proposed mechanisms of PULSAR have been most directly demonstrated, where adaptation resolved a hard heart constraint that the scheduled plan violated in an ultra-central target, and simulation predicted lower lung and heart dose for large central tumours [26,27]. Interval regression reduced dose to serial mediastinal structures at successive pulses in a non-central primary tumour, though lung dose rose in the same case [15]. Central location is also the setting with the best-documented excess toxicity after conventional SBRT [33].	Two case reports of central or ultra-central targets [5,27], one dosimetric simulation [26], one non-central case report contributing per-pulse dosimetry [15], and one recruiting early phase I trial in ultra-central tumours [29]. No comparator.	Feasibility and toxicity, with a prespecified constraint-violation trigger for adaptation and central review of plans
High	Interstitial lung disease, particularly with a reduced baseline DLCO.	ILD was the recorded indication for PULSAR in 29% of the largest cohort and the index comorbidity in one case report, in which no grade 3 or higher event occurred [5,10]. The only post-treatment pulmonary-function signal in the literature comes from a study whose poor-lung-function cohort was defined by moderate-to-severe COPD, restrictive lung disease, or home oxygen use, although the composition of the subset that actually had pulmonary function tests was not reported [24].	One retrospective cohort subgroup [10], one case report [5], one abstract [24]. Certainty very low.	DLCO and FEV1 changes and validated quality of life were assessed as co-primary outcomes, with late toxicity followed to at least two years.
High	Severe COPD or poor pulmonary reserve (GOLD stage 3–4, FEV1 <50% predicted, or clinical assessment of high pulmonary risk).	COPD was the recorded indication in 67% of the largest cohort, that in the study by Westover et al., whose median baseline DLCO was 33% predicted in the 16 patients in whom it was measured and whose one-year overall survival was 74%, which the authors compared favourably with historical rates after conventional SBRT in severe COPD [10].	One retrospective cohort [10] and one abstract [24]. No comparator.	Pulmonary function, oxygen requirement, dyspnea scores, and unplanned respiratory admissions alongside local control.
Moderate to high	Large or bulky thoracic tumours in which substantial interval regression is plausible.	Larger initial tumour volume was associated with greater absolute and proportional shrinkage in the largest cohort, although the authors note substantial variability within size subgroups, and the most striking regression in the literature, from an internal target volume of 24.2 cm^3^ to a low of 4.3 cm^3^ at the third fraction, occurred in a 2.6 cm central tumour, while in a 71 × 49 mm primary tumour, the internal target volume fell by 28% between the first and third adapted pulses [5,10,15]. Regression is the mechanism on which adaptive benefit depends.	One cohort [10], two case reports [5,15], one simulation [26].	Volumetric response as a stratification variable with a prespecified volumetric threshold for adaptation and for omission of later pulse.
Moderate	Reirradiation after prior thoracic radiotherapy.	Prior thoracic irradiation requiring re-treatment was an explicit eligibility criterion in the largest cohort, that in a study by Westover et al., but outcomes for this subgroup were not reported separately.	Subgroup within one cohort, not analyzed separately.	Disaggregated reporting of cumulative dose, interval, and late toxicity before this subgroup can be prioritized further.
Moderate	Oligometastatic NSCLC receiving concurrent immune checkpoint blockade.	This is the setting with the strongest mechanistic rationale, supported by schedule-dependent synergy in murine models [3,8]. However, the single reported case, Eustace et al., developed grade 2 pneumonitis of uncertain attribution [27], and three registered trials combining PULSAR with checkpoint blockade closed with between zero and six participants enrolled [30,31,32].	One case report [27], preclinical models [3,8], and terminated or withdrawn registry records [30,31,32].	Multicentre design with high accrual as a stated constraint, prespecified sequencing of PULSAR and checkpoint blockade, and pneumonitis as a named endpoint.
Lowest (not recommended)	Peripheral, early-stage NSCLC in which a standard SBRT plan meets all constraints.	Conventional SBRT already achieves high local control with low toxicity in this setting [26,34,35] and no reviewed study identified any signal of added benefit from PULSAR. Against this, PULSAR extends treatment to two or three months, carries a risk of interval systemic progression (six of 24 patients progressed systemically in the largest cohort [10]), and imposes a substantial adaptive workload.	No evidence of added benefit in any included. study	Not a priority for PULSAR trials and resources are better directed to the subgroups above.

## Data Availability

No new data were created or analyzed in this study. Data sharing is not applicable to this article.

## Supplementary Materials

The following supporting information can be downloaded at: https://www.mdpi.com/article/10.3390/cancers18183021/s1, Table S1: PRISMA 2020 checklist; Table S2: Search strategy; Table S3: Individual risk-of-bias assessment.

## Abbreviations

The following abbreviations are used in this manuscript: PULSAR, personalized ultrafractionated stereotactic adaptive radiotherapy; SBRT, stereotactic body radiotherapy; SABR, stereotactic ablative radiotherapy; OAR, organ at risk; ICB, immune checkpoint blockade; NSCLC, non-small cell lung cancer; ILD, interstitial lung disease; PRISMA, preferred reporting items for systematic reviews and meta-analyses; SWiM, synthesis without meta-analysis; LLC, Lewis lung carcinoma; α-PD-L1, programmed death ligand-1 blockade; COPD, chronic obstructive pulmonary disease; CNS, central nervous system; ITV, internal target volume; PFS, progression-free survival; DLCO, diffusing capacity of the lungs for carbon monoxide; FEV1, forced expiratory volume in 1 s; MC38, murine colon adenocarcinoma 38; CD, cluster of differentiation; CBCT, cone-beam computed tomography.
