# Supplementary material for: Personalized Ultrafractionated Stereotactic Adaptive Radiotherapy in Lung Cancer and Lung Metastases: A Systematic Review
Source: Cancers (Basel). 2026 Sep 17;18(18):3021. doi: 10.3390/cancers18183021 (PMC13604482; doi:10.3390/cancers18183021)
Supplement: Supplementary file 1 [file cancers-18-03021-s001.zip › Table S2-Search Strategy.pdf]

Table S2. Search strategy.

Medline:
1. exp Lung Neoplasms/
2. ((lung or pulmonary or thoracic) adj3
(cancer* or neoplasm* or carcinoma* or tumor* or tumour* or NSCLC or SCLC)).ti,ab,kw.
3. 1 or 2
4. exp Stereotactic Body Radiotherapy/
5. exp Radiosurgery/
6. ((stereotactic adj3 (body or ablative)) adj3
(radiotherap* or radiation or irradiat*)).ti,ab,kw.
7. (SBRT or SABR).ti,ab,kw.
8. 4 or 5 or 6 or 7
9. (PULSAR or "personalized ultra-fractionated stereotactic adaptive radiotherapy").ti,ab,kw.
10. ((pulsed or pulse-separated or temporally separated or temporally spaced or ultra-fractionated or temporally optimized or personalized) adj5 (stereotactic or ablative) adj5 (radiotherap* or radiation)).ti,ab,kw.
11. ("extended interval SBRT" or "extended intervals between fractions" or "personalized radiotherapy" or "temporally optimized radiotherapy").ti,ab,kw.
12. ((staged or interval) adj3 radiosurger*).ti,ab,kw.
13. 9 or 10 or 11 or 12
14. 3 and 8 and 13
15. limit 14 to english language

Embase:
1. exp lung cancer/
2. ((lung or pulmonary or thoracic) adj3 (cancer* or neoplasm* or carcinoma* or tumor* or tumour* or NSCLC or SCLC)).ti,ab,kw.
3. 1 or 2
4. exp stereotactic body radiotherapy/
5. exp radiosurgery/
6. ((stereotactic adj3 (body or ablative)) adj3
(radiotherap* or radiation or irradiat*)).ti,ab,kw.
7. (SBRT or SABR).ti,ab,kw.
8. 4 or 5 or 6 or 7
9. (PULSAR or "personalized ultra-fractionated stereotactic adaptive radiotherapy").ti,ab,kw.
10. ((pulsed or pulse-separated or temporally separated or temporally spaced or ultra-fractionated or temporally optimized or personalized) adj5 (stereotactic or ablative) adj5 (radiotherap* or radiation)).ti,ab,kw.
11. ("extended interval SBRT" or "extended intervals between fractions" or "personalized radiotherapy" or "temporally optimized radiotherapy").ti,ab,kw.12. ((staged or interval) adj3 radiosurger*).ti,ab,kw.
13. 9 or 10 or 11 or 12
14. 3 and 8 and 13
15. limit 14 to english language

Central:
#1 MeSH descriptor: [Lung Neoplasms] explode all trees
#2 ((lung or pulmonary or thoracic) near/3
(cancer* or neoplasm* or carcinoma* or tumor* or tumour* or NSCLC or SCLC)):ti,ab,kw
#3 #1 OR #2
#4 MeSH descriptor: [Radiosurgery] explode all trees
#5 (SBRT OR SABR OR stereotactic radiotherap* OR radiosurger*):ti,ab,kw
#6 #4 OR #5
#7 (PULSAR OR "personalized ultra-fractionated stereotactic adaptive radiotherapy"):ti,ab,kw
#8 ((pulsed OR pulse-separated OR temporally separated OR temporally spaced OR
ultra-fractionated OR temporally optimized OR personalized) near/5 (stereotactic OR ablative)

near/5 (radiotherap* OR radiation)):ti,ab,kw
#9 ("extended interval SBRT" OR "extended intervals between fractions" OR "personalized radiotherapy" OR "temporally optimized radiotherapy"):ti,ab,kw
#10 ((staged OR interval) near/3 radiosurger*):ti,ab,kw
#11 #7 OR #8 OR #9 OR #10
#12 #3 AND #6 AND #11
